# The influence of *Escherichia coli *cultivation temperature on interferon alpha 2a expression (IFN-α2a)

**DOI:** 10.1186/1753-6561-8-S4-P2

**Published:** 2014-10-01

**Authors:** Fernanda dos Santos Arthuso, Miriam Fussae Suzuki, Nélio Alessandro de Jesus Oliveira, João Ezequiel de Oliveira, Paolo Bartolini, Carlos Roberto Jorge Soares

**Affiliations:** 1 Instituto de Pesquisas Energéticas e Nucleares, IPEN - CNEN/SP, São Paulo, Brazil; 2 Lillehei Heart Institute - University of Minnesota, Minneapolis, MN 55455, USA

## Background

The Hormone Group of IPEN develops researches on recombinant pituitary hormones produced in *Escherichia coli *at laboratory scale, including human growth hormone (hGH) and prolactin (hPRL). The best results were obtained using the λP_L _promoter and the signal peptide DsbA, and so the protein of interest is secreted into the bacterial periplasmic space, in its authentic form without an initial methionine [1,2]. The expression, under control of the λP_L _promoter, regulated by the thermosensitive repressor, is used for large scale production in *E. coli*. Thermal induction presents a great advantage over chemical inducers like IPTG or nalidixic acid, that are expensive and dangerous for manipulators and environment, but it presents the disadvantage that proteins particularly thermolabile like hPRL could suffer proteolysis and aggregation, influencing negatively the production. Considering the limitations on the use of IPTG and the periplasmic secretion, successfully obtained with our hGH expression vector using λP_L _promoter and W3110 strain, we decided to use this system for IFN-α2a production. The goal of this work was the construction of the IFN-α2a vector, obtaining an the IFN-α2a periplasmic expression.

## Methods

The DNA sequences corresponding to the NdeI restriction site, start codon, signal peptide DsbA, cDNA of the IFN-α2a, stop codon, and the restriction site BamHI were inserted into the plasmid, λPL-DsbA-mPRL [3]. This plasmid contains the λP_L _promoter and the gene for ampicillin resistance [1]. The new vector was called λPL-DsbA-IFNα2a. The IFN-α2a sequence was synthesized by the GenScript Corporation (Piscataway - NJ - EUA) in the pUC57 plasmid. The vector obtained was amplified in *E. coli *DH5α strain and then introduced into the expression W3110 cells. After selection, the best clone was used to test different cultivation temperatures (32°C, 35°C, 37°C, and 42°C). Analyses of the periplasmic fluid obtained by osmotic shock based on Western Blotting and SDS-PAGE were carried out.

## Results and conclusions

Construction of λPL-DsbA-IFNα2a plasmid was confirmed by restriction analysis. IFN-α2a secreted into the periplasmic space of *E. coli *was obtained, as shown in Western Blotting and SDS-PAGE. We can observe that the best growth temperature for the IFN-α2a was 37°C probably because at this temperature the bacteria presented a better growth and the production of aggregates was lower. The production yield was of ~ 0,1 µg/ml/A_600_.
